# Use of Inertial Measurement Units for Detection of the Support Phases in Discus Throwing

**DOI:** 10.3390/s25196095

**Published:** 2025-10-03

**Authors:** José Sánchez-Moreno, David Moreno-Salinas, Juan Carlos Álvarez-Ortiz

**Affiliations:** 1 Department of Computer Science and Automatic Control, Universidad Nacional de Educación a Distancia, 28040 Madrid, Spain; dmoreno@dia.uned.es; 2 Real Federación Española de Atletismo, 28223 Pozuelo de Alarcón, Spain; jcalvarez@rfea.es

**Keywords:** discus throwing, IMU, biomechanics, motion analysis, sport performance monitoring, sports engineering, athletic training technology

## Abstract

Photogrammetry applied to sports provides precise data on athlete positions and time instants, especially with digital motion capture systems. However, detecting and identifying specific events in athletic movements such as discus throwing can be challenging when using only images. For example, with high-speed video, it is difficult to pinpoint the exact frame when events like foot touchdown or takeoff occur, as contact between shoe and ground may span several frames. Inertial measurement units (IMUs) can detect maxima and minima in linear accelerations and angular velocities, helping to accurately determine these specific events in throwing movements. As a result, comparing photogrammetry data with IMU data becomes challenging because of the differences in the methods used to detect events. Even if comparisons can be made with IMU data from other sports researchers, variations in methodologies can invalidate the comparison. To address this, the paper proposes a simple methodology for detecting the five phases of a discus throw using three IMUs located on the thrower’s wrist and on the instep or ankle of the feet. Experiments with three elite male discus throwers are conducted and the results are compared with existing data in the literature. The findings demonstrate that the proposed methodology is effective (100% of phases detected in the experiments without false positives) and reliable (results validated with professional coaches), offering a practical and time- and cost-effective solution for accurately detecting key moments in athletic movements.

## 1. Introduction

The enhancement of training techniques applied to any discipline related to athletics is a process that never stops; especially in the context of high performance, where beating the best records, whether nationally or internationally, is the main objective. In order to achieve these improvements, it has always been necessary to use the most appropriate technology available at that time [1,2]. The best-known example in the arena of track and field throwing events (i.e., discus throw, hammer throw, javelin throw, and shot put) is the continuous evolution of the use of images to analyze the athlete’s technique and try to refine and enhance it [3,4,5,6,7,8,9]. Another recent example is the use of inertial measurement units (IMUs) located at specific points on the athlete’s body [10,11,12] or inside the implement [13,14,15]. IMUs are a type of sensor especially well-suited to the requirements of athletics disciplines [16,17,18,19] that allow very accurate measurements of the linear and angular accelerations of specific parts of the athlete’s body. They also facilitate determining their orientations and deriving directly or indirectly biomechanical parameters (centers of mass, angular momentum, linear velocity) that are relevant to sports researchers or coaches.

However, the great drawback is the significant expense of these technologies, especially for application in elite athletics (such as high-speed synchronized cameras, laser arrays, UWB systems, etc.), which often require advanced hardware/software knowledge to, for example, develop a final application based on a set of IMUs. Moreover, it is important to note that, in many cases, the availability of these systems is restricted to specific high-performance centers or advanced biomechanics laboratories; making it impossible for coaches and athletes, typically located on athletics tracks of universities or cities, to have access to these resources daily. Fortunately, advances in technology such as the miniaturization of electromechanical devices, the increase in computing capabilities and storage space of mobile devices, together with advances in data processing techniques, have paved the way to overcome these limitations and to achieve significant progress in the field. It is now possible to acquire low-cost IMU-based environments focused specifically on capturing kinematic parameters of the human body (e.g., Movella, MbientLab, Opal APDM). Their implementation is practically immediate, without the need for hardware knowledge and with very minimal effort at software level. This latter fact is due to the existence of development libraries and applications for their management through Bluetooth Low Energy (BLE). Furthermore, these environments let practitioners export the results in file formats compatible with specific biomechanical (Visual3D) or scientific (Matlab 2024b) software packages.

Focusing the narrative on the use of IMUs in the discus throw discipline, there are already some works that deal with the measurement of angles and orientations of both the implement [13] and specific parts of the thrower’s body [10,20,21]. However, to the best of the authors’ knowledge, there is a lack of literature for detecting the five stages or phases of a discus throw [5] using IMUs, all the references found are based on the analysis of video recordings [6,7,20,22,23]. In these references, the duration of each phase was extracted by replaying the recorded attempts of the participants frame by frame and using different frame rates and data processing techniques to detect the body landmarks.

Although the scientific studies on the correlation between duration of the phases and throw distance have produced contradictory results, as can be observed in [6,22,24], the time spent on each phase of discus throw indicates the athlete’s movement coordination and enables us to analyze the evolution of the Z and Y angular momenta generated in the thrower, in the discus, and in the thrower-plus-discus systems [25,26]. This underscores the need for further research on thrower movements within the circle using recent and affordable technologies.

**Figure 1:**
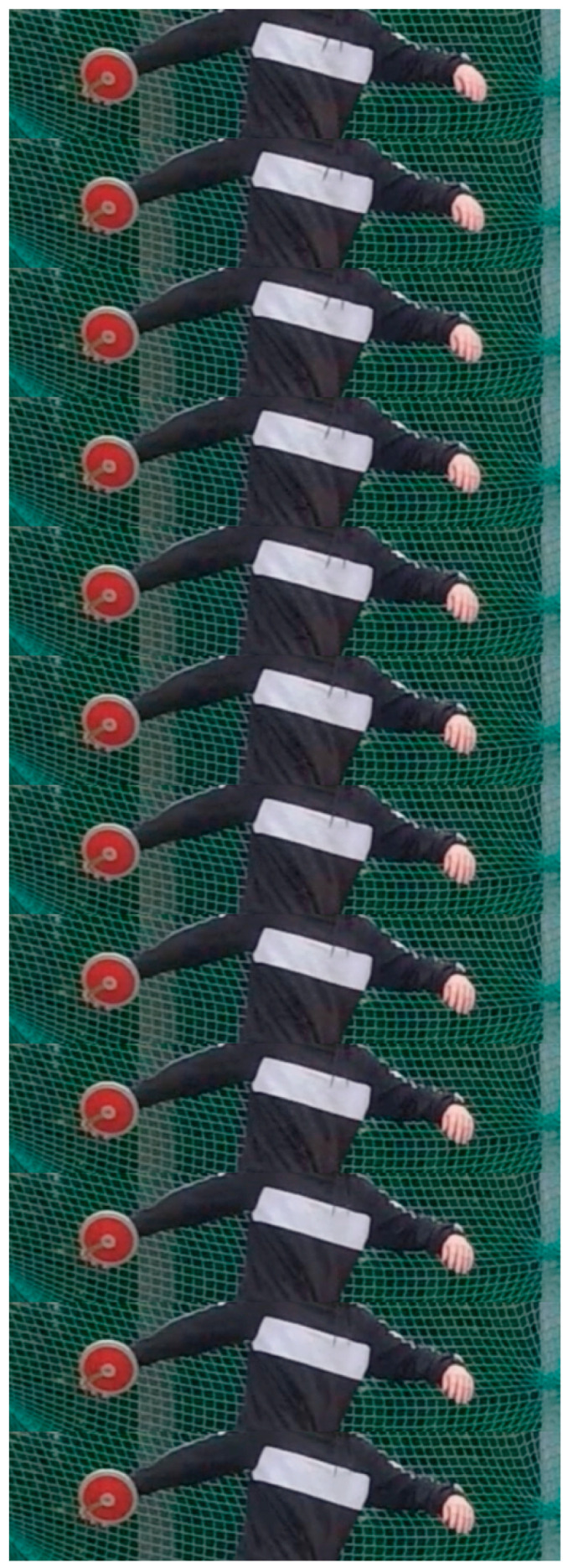
Sequence of 12 frames from a 120-fps video recording to detect the time instant corresponding to the maximum backswing of the thrower arm, which determines the beginning of the throw.

Analyzing the movement patterns of Spanish elite discus throwers at the High-Performance Sports Centre (CAR) in Madrid, we observed that the duration of the phases obtained by IMUs and by video tools (mobile phones or tablets recording at 120/200 fps and Kinovea, a free video annotation tool designed for sports analysis) accessible to coaches during routine training sessions differed. Two reasons were identified: (1) it is difficult to pinpoint the exact video frame in which an event occurs (for example, the release of the discus, as it is not clear when the discus is no longer being pushed by the hand), and (2) the cushioning of shoes and foot soles can result in false visual positives, which means that a shoe can be in visual contact with the floor along several frames, but it is impossible to determine whether the athlete’s full weight is pushing the ground. This problem was previously identified by [20], who stated: “In the case of a critical instant being difficult to determine because foot contact was obscured or the image was blurred, the critical instant was not included in the synchronization procedure.” As an example of the above-mentioned problems in analyzing a throw, Figure 1 presents a sequence of 12 consecutive frames of a throw recorded at 120 fps (the total sequence is composed of 181 frames, that is, 1.508 s.) in which it is nearly impossible to pinpoint the exact frame that corresponds to the maximum backswing of the athlete’s arm. It should be noted that a second camera located behind the athlete would not have simplified the problem, as the view of the discus movement would have been similar but from another perspective, and increasing the capture rate would have only produced more frames with the same uncertainties.

For these reasons, it is not entirely fair to compare data obtained by IMUs with data obtained by video analysis. Additionally, as there is no research literature about the use of IMUs to detect the phases of a discus throw, it is necessary to share a procedure to provide sport researchers, coaches, and athletes with a reference for technique improvement.

The main purpose of this study is to propose a methodology for determining discus throw phase durations using commercial IMU data. We hypothesized that the phases duration of a discus throw cannot be exactly obtained only by video recordings due to the previously exposed reasons. We also hypothesized that IMUs let us determine with accuracy the time instants when the events that delimit the phases occur (that is, the maximal backswing of the throwing arm, the takeoffs and touchdowns of the feet, and the release of the discus). This way the IMU-based procedure described in the paper may serve as a common framework for comparison purposes if other sport biomechanicists or coaches decide to employ the IMU technology to analyze the movements of discus throwers. Thus, if these hypotheses are fulfilled the procedure would become a novel and additional tool for sports biomechanicists, as well as of major interest for coaches and throwers.

## 2. Methods

### 2.1. Participants

The participants in this study were three Spanish male elite discus throwers, who regularly throw a 2 kg discus more than 55 m in training sessions. The basic information regarding the athletes is presented in Table 1. All participants were right-handed, had been informed of the purpose of this study, and had provided their informed consent.

### 2.2. Equipment and Data Collection

**Figure 2:**
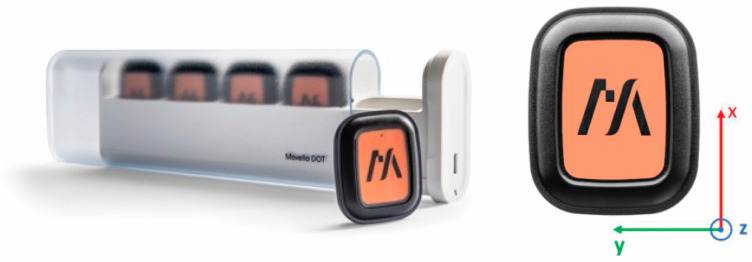
Kit of Movella DOTs (**left**) and front view of an IMU (**right**) with its local reference system.

The Movella DOT development platform [26] was selected as the inertial measurement system (Figure 2). This consumer-grade product consists of a set of five 9-axis IMUs (accelerometer + gyroscope + compass), called DOTs, and specifically designed to record kinematic data from the human body up to a maximum frequency of 120 Hz. This product was selected due to its advantages over other commercial IMUs: it does not require pre-calibration, and the time synchronization of the units, the setup, the start/stop of the recordings, and the exporting of data are user-friendly, making it accessible for people without technical profiles. This feature is particularly important from the point of view of coaches and athletes, who are the true final users.

**Figure 3:**
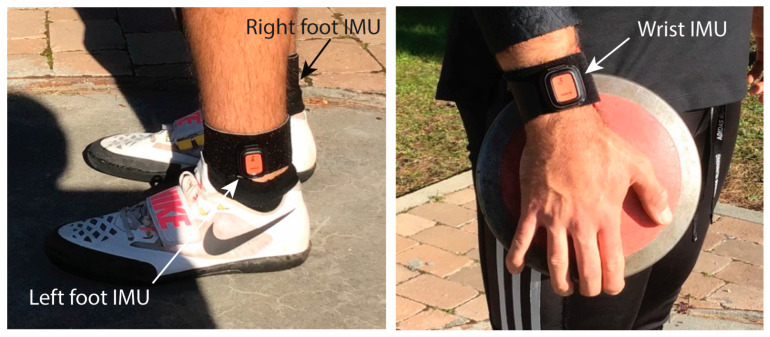
Location of the three IMUs on the athlete body. The IMU on the right ankle is hidden in the photo.

The setup employed with the athletes consisted of three DOTs attached to the right wrist (with the *X*-axis pointing to the shoulder and the *Y*-axis to the back) and to the left and right ankles (with the *X*-axis pointing to the head and the *Z*-axis pointing outwards from the body in both cases). Figure 3 presents the location of the IMUs. The three IMUs (and an additional IMU installed temporarily inside the discus) were always setup to sample at 120 Hz, and the magnitudes recorded were linear accelerations (in m ⋅ s^−2^), angular velocities (in deg ⋅ s^−1^), and Euler angles (in deg). The performance ranging of the accelerometer, gyroscope, and magnetometer of the IMUs are fixed by the manufacturer to ±16 g, ±2000°/s, and ±8 Gauss, respectively. By default, the DOTs’ local earth-fixed reference coordinate system is defined as a right-handed Cartesian coordinate system with X positive toward East (E), Y positive toward North (N), and Z positive when pointing up (U). The IMUs incorporate an internal calibration algorithm based on strapdown integration (SDI) and Kalman filtering. The Kalman filter uses the output from the SDI to compute a statistically optimal 3D orientation estimate of high accuracy, with no drift for both static and dynamic movements. However, because the discus cage located around the athlete contains ferromagnetic materials, the magnetic field can become distorted, causing errors in the measured orientation. To correct these magnetic disturbances and compensate for the distorted magnetic field, a calibration of the IMU’s magnetometer must be performed when the training location changes (for example, when moving to another track and field facility).

All data were collected at the outdoor track and field training facilities located at the headquarters of the CAR in Madrid using a competition standard discus throwing circle and a 2 kg homologated implement. All the trials measured and presented in this paper were recorded without integrating an IMU or any other sensor inside the discus (as will be explained in the next section, an IMU was introduced in the discus but only temporarily, not during the trials reported in the paper). The throws were recorded in two training sessions for each athlete during his yearly training plan (a total of six sessions) and the throwing distance was not measured. After each experiment, the data stored in each IMU were exported in csv format and imported into Matlab 2024b for their processing as described in Section 2.4.

It is important to remark that the IMUs are initialized and synchronized by software, using the same internal clock for all IMUs. Due to communication constraints and delays, it is possible that IMUs do not start recording at the same sampling time, with a possible offset of one sampling time (0.0833 s) between signals. This can be easily corrected with a simple pre-processing phase, considering the common clock and that the data sequence to be analyzed begins at the moment when the three IMUs transmit simultaneously for the first time. Regarding this preprocessing stage, it was not necessary to filter the raw signals as the IMU firmware incorporates filtering to provide cleaner and more accurate motion data. The type of filtering can be selected via software, and for the present work, the dynamic filtering was selected, which is based on the Kalman filter and, as indicated in the documentation, is designed for its application on persons for sports. The use of additional filter in the preprocessing stage, although tested in the initial versions of the algorithm, did not provide any additional advantage.

In addition, a video camera recording at 120 frames per second (the same sampling rate of the DOTs) and positioned on the left side of the athlete captured each throw. The video recorders used were smartphones, tablets (Apple (Cupertino, CA, USA), Samsung (Seoul, Republic of Korea)), or GoPro cameras (San Mateo, CA, USA). No pre-calibration work was carried out because the critical temporal instants were obtained by a frame-to-frame visual inspection of the position of feet and discus in the recording, using the video annotation tool Kinovea to register the timing and were compared with the information obtained by the IMUs. These video recordings and the analyses to obtain the critical temporal instants were carried out using hardware and software that are easily accessible to any coach or athlete. The idea was to generate information in a similar way that any coach and athlete could afford at home without special equipment.

### 2.3. Phases of a Discus Throw

There is a consensus among researchers on biomechanics and trainers on discus throw, that the technique currently employed by athletes is divided into five phases [5,6,7,23,27]. The names of each of the five phases and their temporal definitions are (for a right-handed thrower):

The preparation or double-support phase: it is defined from the instant of maximum backswing of the throwing arm, *t_start_*, that establishes the start of the throw to the instant that the right foot leaves the ground, *t_takeoff_right_*.

The initial, entry, or first single-support phase: it goes from the instant that the right foot leaves the ground, *t_takeoff_right_*, to the instant that the left foot leaves the ground, *t_takeoff_left_*.

The non-support, airborne, or flight phase: from the instant that the left foot leaves the ground, *t_takeoff_left_*, to the instant that the right foot lands, *t_touchdown_right_*.

The transition or second single-support phase: from the instant that the right foot lands, *t_touchdown_right_*, to the instant that the left foot lands, *t_touchdown_left_*.

The delivery phase: from the instant that the left foot lands, *t_touchdown_left_*, to the instant that the discus is released from the participant’s hand, *t_release_*.

**Figure 4:**
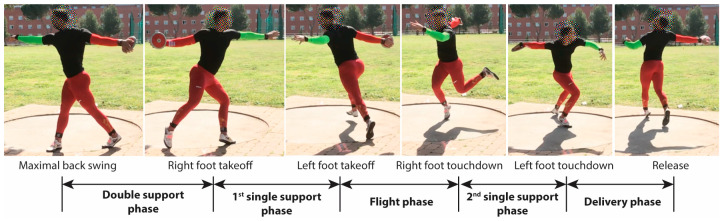
Critical instants and phases of discus throw captured during a trial in the athletic facilities of CAR in Madrid.

**Figure 5:**
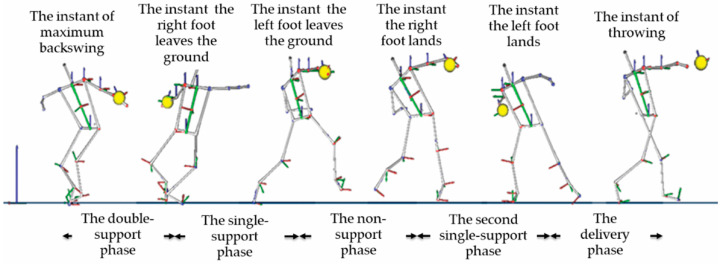
Critical instants and phases of discus throw defined in the literature. Source: [6].

According to the temporal definition of these five phases, there are six critical temporal instants or events that must be detected. In the literature and for comparison purposes, the duration of the five phases is calculated as the ratio of each phase’s duration to the total throw time. Figure 4 and Figure 5 present a photo and a graphical sequence of the phases.

### 2.4. Procedure for Detecting the Phases of the Discus Throw

To detect the six temporal events using IMUs, first it is necessary to separate the detection of *t_start_* and *t_release_* from the four events related to the feet. These two temporal limits of a throw are obtained by the IMU located at the wrist.

#### 2.4.1. Detection of the End of the Movement

The release of the discus is considered to occur at the time instant that the discus experiences maximum free acceleration (that is, maximum acceleration in the local Earth coordinate system). Free acceleration is defined as the acceleration in the local earth-fixed coordinate system, from which the local gravity is subtracted.

**Figure 6:**
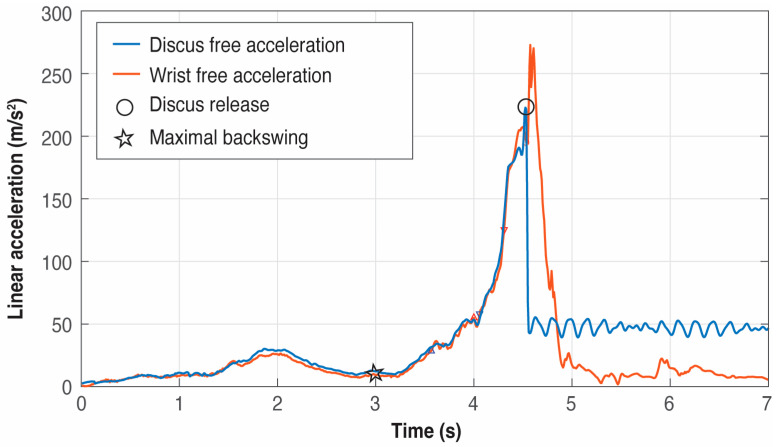
Example of free acceleration curves and their differences between the discus (blue) and the wrist (red) in a trial using an IMU temporarily embedded in the discus.

Notice that in a throw, the free acceleration of the discus suddenly decreases at the moment of its release from the hand [13], but its maximum always occurs in a previous time instant to the maximum of the wrist acceleration (see Figure 6). However, although the setup of the procedure explained in the paper does not need an IMU inside the discus (also, it is forbidden to introduce an IMU within the implement in an official competition), it was temporarily necessary to embed a DOT inside a discus in order to correctly validate the release time instant.

**Figure 7:**
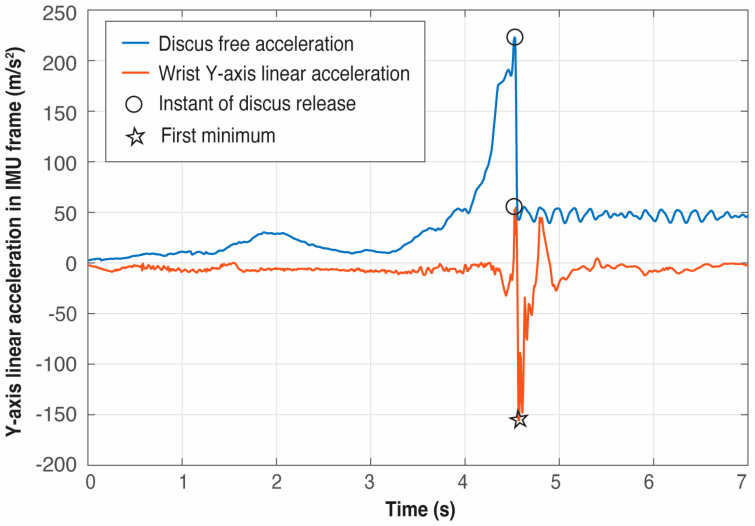
Example of detection of the release of the discus using the data from the IMU located at the wrist (blue), and compared with the data from the IMU temporarily embedded in the discus (red).

The purpose of this provisional DOT was to make preliminary experiments to register the time instant of the maximum free acceleration in the discus and to look for a simultaneous and well-defined event (that is, a maximum or a minimum) in some of the acceleration signals in the local coordinate system of the wrist IMU. This method allowed us to indirectly determine the end of a throw with just the wrist IMU, that is, to identify a proxy to be used instead of the acceleration data provided by a discus IMU. After several trials and analysis of the local accelerations of the wrist IMU, it was detected that the discus release happened at the global maximum located before the global minimum in the Y-axis linear acceleration of the wrist IMU (see Figure 7). Once this event in the IMU wrist was identified as the proxy to measure the release of the discus, the IMU inside the discus was definitively removed.

The comprehensive analysis of all trials showed that the first minimum in the Y-axis of the linear acceleration of the wrist after *t_release_* is indeed a global minimum in the signal and allows us to unambiguously fix the point to look backward for the global maximum that indirectly defines *t_release_*.

#### 2.4.2. Detection of the Start of the Movement

Once *t_release_* is known, it is needed to look backwards to find *t_start_*. This event is detected as the time instant at which the throwing arm changes its direction by examining its path from the end of the movement to the beginning. This is achieved by analyzing the Z-angle trace of the wrist IMU in a reverse way, that is, from *t_release_* until a change in sense is detected, that is, until a local minimum is found. This first minimum corresponds to the maximum backswing of the throwing arm. It should be noted that the angle trace in degrees of the hand on the *Z*-axis is calculated considering that the initial yaw is 0° at the time when the wrist IMU starts recording. Thus, the absolute path in Z is generated by accumulating the differences in degrees between two consecutive measurements of the IMU.

The internal algorithms of the IMUs work with quaternions, avoiding gimbal lock problems, and can provide orientation measurements both as quaternions and as Euler angles. For the sake of visualization, the Euler option was chosen. Since only the rotation around the *Z*-axis from the wrist IMU is used and the motion takes place in a horizontal plane parallel to the ground, gimbal lock is not an issue, even in the event of using Euler angles.

The range of the Z-angle is [−180°, 180°], and an angle unwrapping algorithm was implemented, which converts discontinuous Euler angle data into continuous measurements by detecting and correcting wrap-around jumps. It iterates through the angle sequence and identifies sudden jumps larger than 350° between consecutive samples, which indicate wrap-around events (e.g., transitions from +179° to −179°). When detected, it calculates a cumulative offset by adding or subtracting 360° in the opposite direction of the jump, and then applies this correction to create a smooth, continuous angular progression. This is essential for discus throw analysis because athletes perform multiple rotations exceeding 360°, and without unwrapping, the raw Euler angles would show artificial discontinuities that would corrupt velocity calculations and phase detection algorithms.

**Figure 8:**
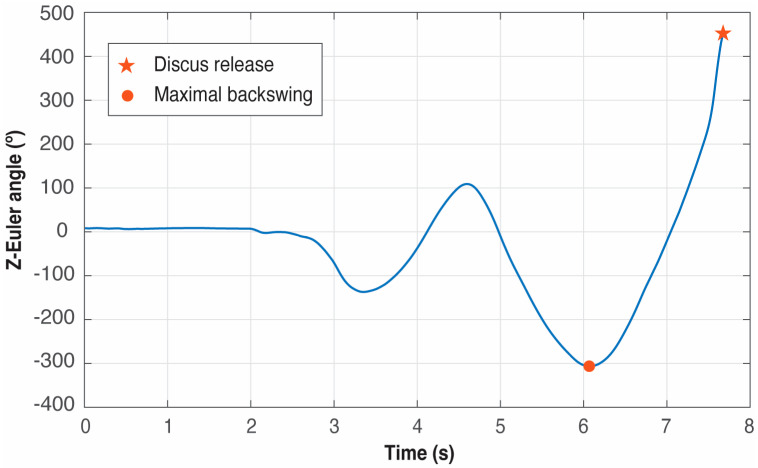
Example of detection of the start of the movement using the Z-Euler angle of the IMU located at the wrist. The red dot represents *t_start_*, that is, the time instant when the maximal backswing of the discus happens.

Figure 8 presents an example. It must be noticed that trying to detect the start of the movement before the end can produce false positives because the initial pre-swings of the athletes with the throwing arm to generate momentum produce several local minima in the Z-Euler angle signal (see Figure 8), and in some trials even a global minimum can appear before the minimum corresponding to the start of the movement. If the search for the minimum is started backward from *t_release_*, the first minimum found corresponds unequivocally to *t_start_*.

Once the start and end instant times are known, the duration of the throw is clearly defined as *t_duration_*= *t_release_ − t_start_.*

#### 2.4.3. Detection of the Events Associated with the Movement of the Feet

With the movement delimited by *t_start_* and *t_release_*, the next step is to identify the four events associated with the feet inside this temporal window. This sequence of detection of the events is very important because if the experiment is explored without considering the temporal window delimited by *t_start_* and *t_release_*, false positives may be detected since there are maxima and minima produced by the vertical movements of the athlete before the start of the true throwing movement (that is, *t_start_*) and after the discus release (that is, *t_release_*). These false positives would correspond to actions without biomechanic and kinematic significance.

Due to the nature of the feet movement, the variable to be analyzed must be the *Z*-axis acceleration in the local Earth coordinate system, that is, the acceleration of the feet in the vertical direction.

With the temporal window of the movement already identified, it is key to detect firstly the global minimum in the *Z*-axis of the left foot, that is, *t_reference_* (that corresponds to the time when the left foot starts descending and the right foot is descending too—the non-support phase) and then look backward until *t_start_* for the two global maxima that define both takeoffs. Thus, the takeoff instants of the right and left foot, *t_takeoff_right_* and *t_takeoff_left_*, correspond to the first maxima before *t_reference_*.

Once the feet are in the air, identifying the two landings is straightforward because they are defined by the global maximum between *t_takeoff_rigth_* and *t_release,_* and *t_takeoff_left_* and *t_release_*, respectively. Thus, the following maximum in the acceleration signal of the right foot from *t_takeoff_left_* is the touchdown instant *t_touchdown_right_*. And the subsequent maximum in the left foot acceleration from *t_touchdown_right_* defines the left touchdown instant *t_touchdown_left_*.

**Figure 9:**
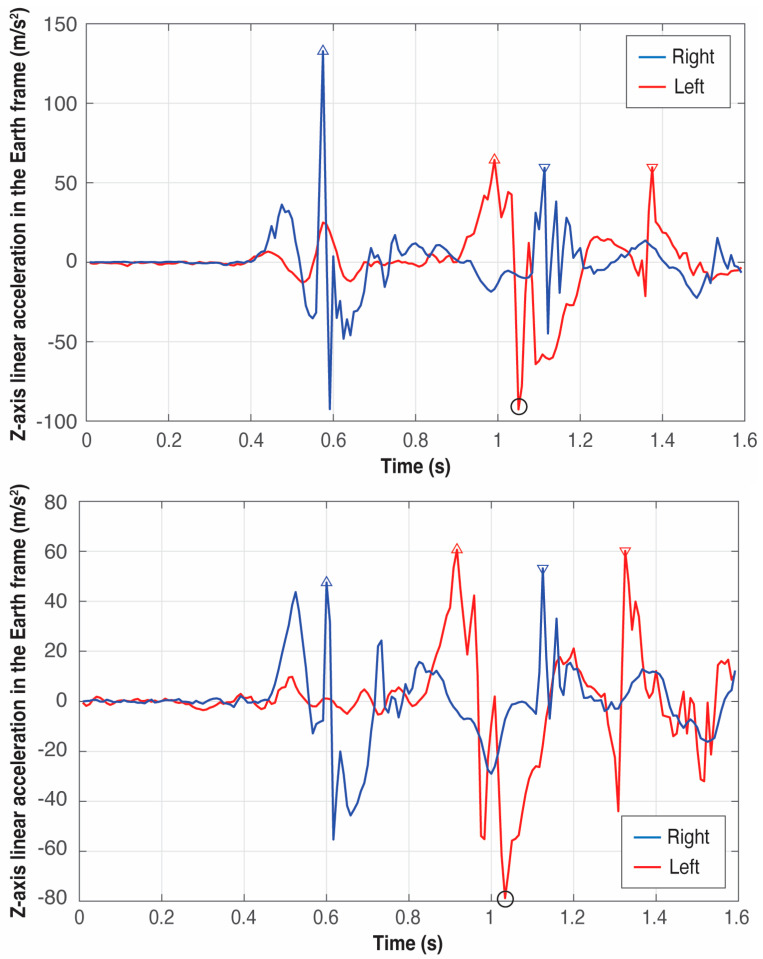
Detection of the four critical events of the left (red) and right (blue) foot in two trials of athlete A. The triangles pointing upwards represent the takeoff of the feet and the triangles pointing downwards represent the landing. The circle in black represents the global minimum in the *Z*-axis of the left foot used to define the four critical events.

**Figure 10:**
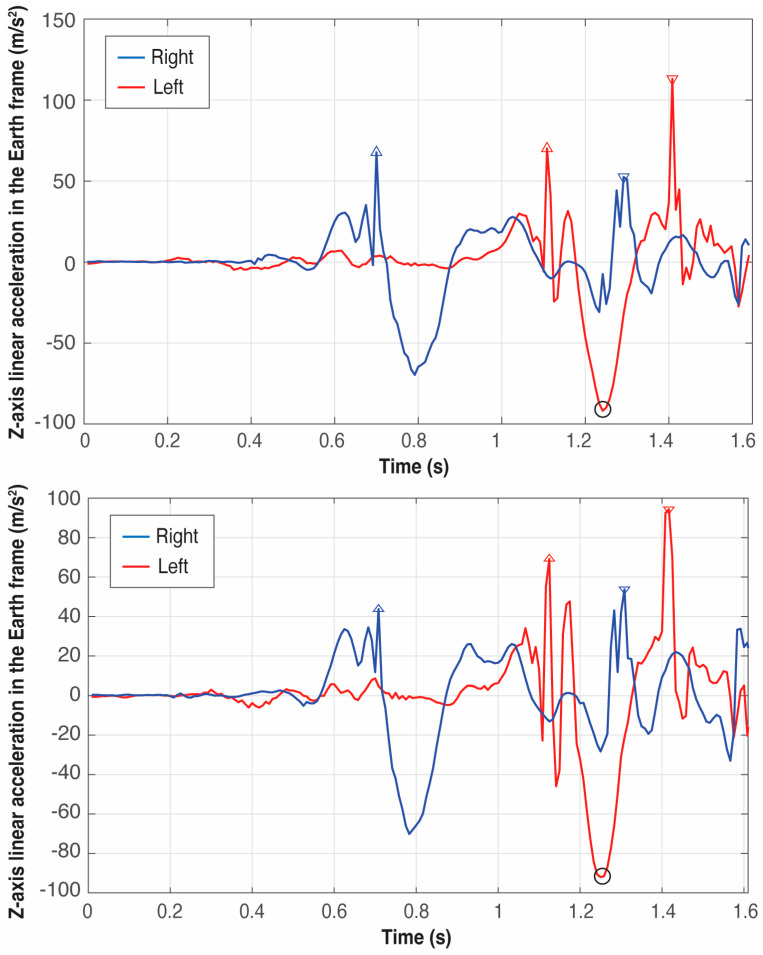
Detection of the four critical events of the left (red) and right (blue) foot in two trials of athlete B. The triangles pointing upwards represent the takeoff of the feet and the triangles pointing downwards represent the landing. The circle in black represents the global minimum in the *Z*-axis of the left foot used to define the four critical events.

**Figure 11:**
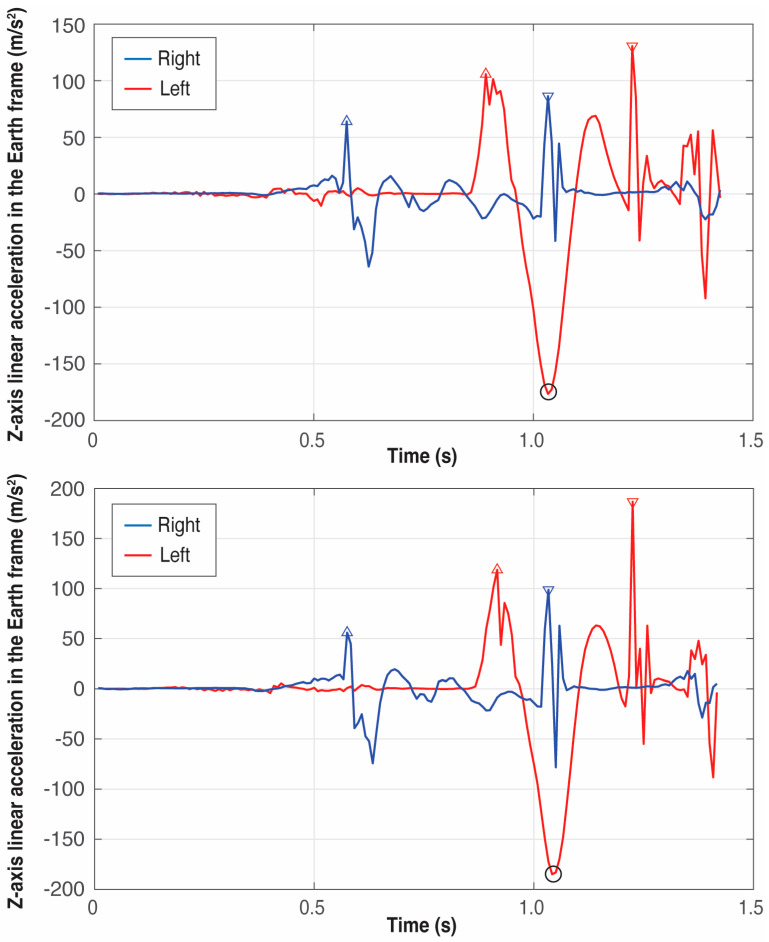
Detection of the four critical events of the left (red) and right (blue) foot in two trials of athlete C. The triangles pointing upwards represent the takeoff of the feet and the triangles pointing downwards represent the landing. The circle in black represents the global minimum in the *Z*-axis of the left foot used to define the four critical events.

Figure 9, Figure 10 and Figure 11 present six examples of the events detection of three athletes (see Table 1). As the examples correspond to different throwers, the difference between peaks can appear quite large in some cases while in others, they look quite similar. There are not two identical throws even for the same athlete, but the pattern of the signals generated by an athlete is always very repetitive, especially at this level of expertise.

An example of the importance of identifying firstly *t_reference_*, that is, the global minimum in the *Z*-axis of the left foot, which can be observed by comparing the right foot patterns of athletes B and C in Figure 10 and Figure 11, respectively: athlete B presents the global maximum of the right foot at the takeoff; however, athlete’s C global maximum occurs at the touchdown. Thus, the event associated with the global minimum in the *Z*-axis of the left foot is the boundary between these two maxima.

In the case of a left-handed thrower, the procedure would be very similar, simply changing the order of detection of the foot patterns and looking for the maximum on the *Z*-axis of the right rather than the left foot to delimit the takeoff and landing times.

**Figure 12:**
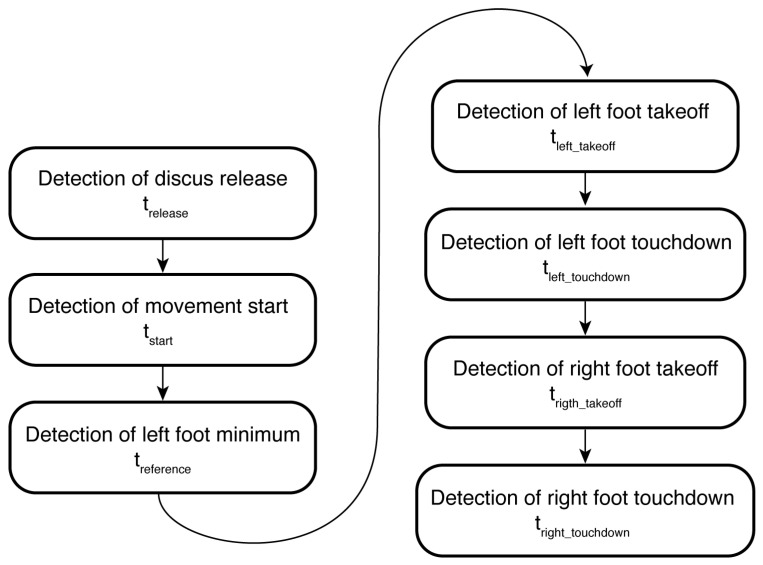
Flowchart with the sequence of detections.

Figure 12 presents a flowchart with the order of detection of the temporal events needed to determine the duration of the five phases that compose a discus throw. Also Listing 1 presents an excerpt of the Matlab code using a multi-step process for detecting the temporal events. First, it determines the discus release time by locating the global minimum in Y_linearAccelerationWrist. The maximum acceleration that occurs before this minimum point establishes the discusDelivery time instant. Next, the beginning of the movement is detected by analyzing backwards the signal Z_anglePathWrist from the time instant discusDelivery to identify the first local minimum where the signal changes from decreasing to increasing values, defining startMovement. By using these two temporal boundaries (from startMovement to discusDelivery), the Z_accelerationLeftFoot and Z_accelerationRightFoot data are analyzed. A reference point is determined by finding the global minimum in Z_accelerationLeftFoot, which allows us to identify the takeoff and touchdown events for the feet: takeOffLeftFoot and touchDownLeftFoot are determined by locating maximum acceleration peaks before and after the reference point in Z_accelerationLeftFoot, while takeOffRightFoot and touchDownRightFoot are identified using the same approach in Z_accelerationRightFoot within the temporal constraints defined by the left foot events.
**Listing 1.** Excerpt of the Matlab code for detecting the temporal events.1 2 3 4 5 6 7 8 9 10 11 12 13 14 15 16 17 18 19 20 21 22 23 24 25 26 27 28 29 30 31 32 33 34 35 36 37 38 39 40% Looking the minimum in the *Y*-axis linear acceleration of the wrist [~,aux1] = min(Y_linearAccelerationWrist); % Looking for the first maximum [~,aux2] = max(Y_linearAccelerationWrist(1:aux1)); discusDelivery = aux1 – 1 + aux2;
% Looking for the first minimum from the discus delivery time % Not using the min() function because the minimum cannot be global data_old = Z_anglePathWrist(discusDelivery); for ii = discusDelivery-1:–1:1 aux = Z_anglePathWrist(ii); if (aux-data_old > 0) startMovement = ii + 1; break; end data_old = aux; end
% Using just the data of the movement Z_accelerationLeftFoot = Z_accelerationLeftFoot(startMovement:discusDelivery); Z_accelerationRightFoot = Z_accelerationRightFoot(startMovement:discusDelivery);
% Looking for the global minimum in the *Z*-axis of the left foot as reference [~,reference] = min(Z_accelerationLeftFoot); 
% Takeoff left foot [~,delta] = max(Z_accelerationLeftFoot(1:reference)); takeOffLeftFoot = delta;
% Touchdown left foot [~,delta] = max(Z_accelerationLeftFoot(reference:end)); touchDownLeftFoot = reference+delta-1;
% Takeoff right foot [~,delta] = max(Z_accelerationRightFoot(1:takeOffLeftFoot)); takeOffRightFoot = delta;
% Touchdown right foot [~,delta] = max(Z_accelerationRightFoot(takeOffLeftFoot:touchDownLeftFoot)); touchDownRightFoot = takeOffLeftFoot+delta-1;

## 3. Results

### 3.1. Measurements of the Trials

**Figure 13:**
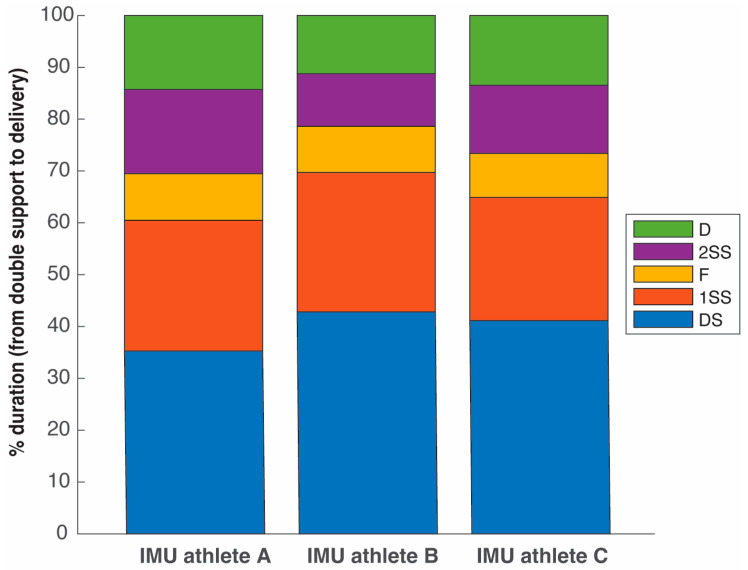
Comparison of the five phases between the three Spanish athletes. The duration of the double support (DS), the first single-support (1SS), the flight (F), the second single-support (2SS), and the delivery (D) phase are expressed as a percentage of the total duration from DS to D.

Table 2 and Figure 13 present the temporal analyses of the Spanish athletes (described in Table 1) applying the IMU-based procedure outlined in previous sections. Note that this procedure has been incorporated into a Matlab program, described in Listing 1, for the automatic detection of the time events of interest using the data provided by the IMUs.

While there are differences between the three athletes, the data demonstrate intra-thrower consistency for the durations of the separate phases. Athlete C shows the highest consistency in his movement (SD < 1% in all the phases), probably due to his higher proficiency (PB, age) and performance state (his experiments were conducted at the end of winter 2025). Athlete A shows the highest variability in the phases, which may be attributed to his expertise (the younger) and that trials were recorded at the beginning of his training cycle (autumn 2024) when the athlete’s performance was lower. All the experiments were programmed after regular training sessions, rather than in official competitions.

### 3.2. Comparison Between Data from IMU and Data Extracted from the Literature

As stated in the introduction, all the research studies on the temporal breakdown of the five phases are based on data extracted from video recordings, using one or two cameras. The six temporal events and the body landmarks are detected using different motion analysis software packages (for example, V1 Home, Kwon3D, Signal TEC3D-Video, or Motus). If markers attached to the athlete joins are used, the 3D coordinates in the global reference frame can be obtained by transformations (Direct Linear Transformation), interpolations (cubic or quintic splines), and filtering (fourth-order recursive low pass, for instance) methods using the motion analysis suites. It should be noted that all of these video-based analyses require meticulous pre-calibration work (identification of global reference markers to define a calibration volume, local control points on the body, fixed locations of the cameras with respect to the discus throwing circle, synchronization of video recordings, etc.). Additionally, the post-processing stage requires some expertise in signal processing and statistics. This process can be both very time-consuming and technically complex for coaches and athletes.

Comparison and validation of the results in Table 2 and Figure 13 with data from other sources are challenging due to the different technologies applied. However, as far as the authors know, there are not similar works to compare with due to the novelty of the methodology proposed, in which the phases are detected using IMUs attached to the athletes. Then, the comparison with literature data can only be made against these sources, which use visual inspection and video analysis. The aim of this comparison is not the statistical validation of the methodology proposed compared with literature, but to show that the results obtained are in line with those obtained with other equipment usually used in professional athletics.

**Figure 14:**
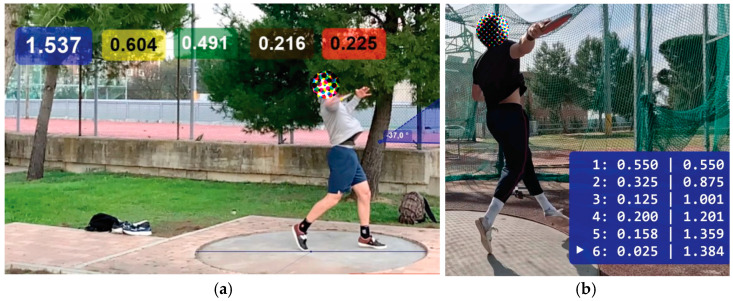
Two examples of detection of critical temporal instants during a trial by a professional coach using an Apple smartphone (**a**) and a GoPro camera (**b**) as video recorders and Kinovea for the labeling.

**Figure 15:**
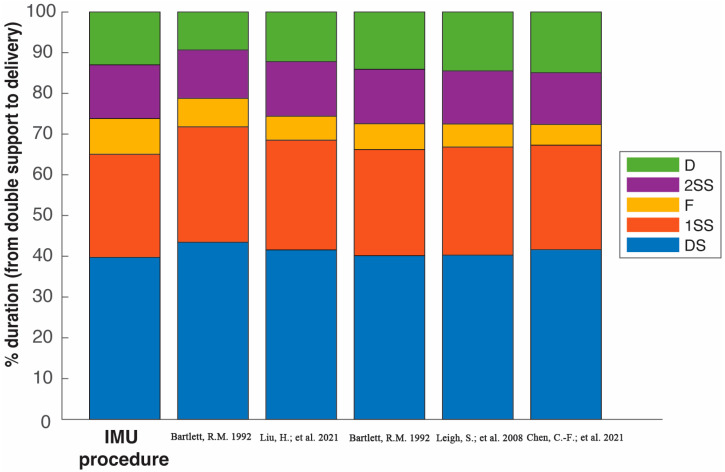
Comparison of the five phases between the Spanish and world elite male discus athletes described in references of Table 3. The duration of the double support (DS), the first single-support (1SS), the flight (F), the second single-support (2SS), and the delivery (D) phase are expressed as a percentage of the total duration from DS to D [6,20,22,23].

In these sources (see Table 3), the critical instants are annotated by visual inspection of the frames (see Figure 14). As outlined in Section 1, determining the foot contact by analyzing frame by frame can be difficult. However, the comparison must be made, and it is presented in Figure 15 using data from elite male athletes obtained from five sources.

### 3.3. Comparison Between Data from IMUs and from Video Recordings

**Figure 16:**
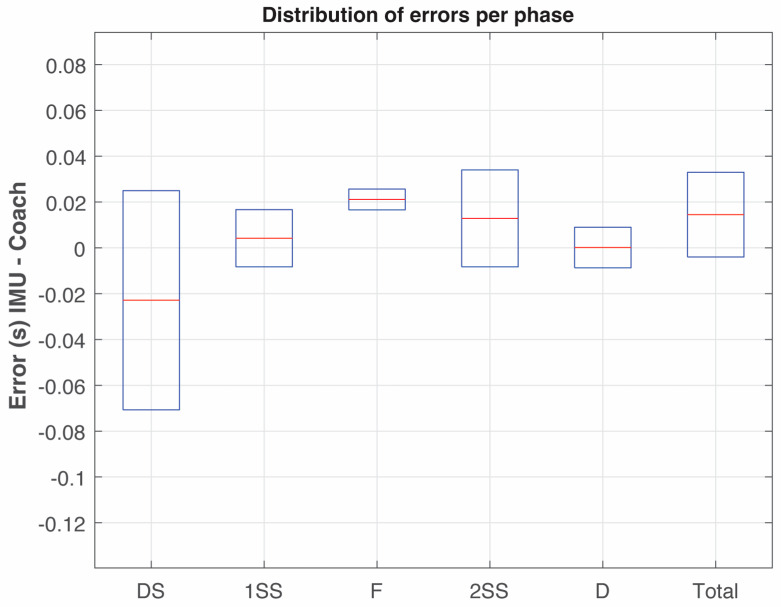
Distribution of IMU-coach differences (errors) across phases. Boxes represent the interquartile range (IQR), horizontal line = median.

For the sake of clarity and for comparison purposes, the duration of the phases of two throws by athletes A and C are shown in Table 4 and Figure 14. These were measured using IMUs and by a professional coach using video recordings. The frame selected by the coach was determined by his/her own experience, which was to select the mid-frame in case the coach had doubts about the exact frame in which the event occurred. The frame selection and event determination by the coach were made without any knowledge of the IMU measurements. In addition, a quantitative and statistical analysis is presented in Table 5, and in Figure 16 the distribution of errors per phase between the coach and IMU measurements is shown.

The resolution lower bound for both methods, given the sampling frequency, is of ±0.5 frame ≈ ±4.17 ms. The mean bias (IMU-Coach) and its 95% confidence interval (CI95) quantify the average systematic difference between methods. The Bland–Altman limits of agreement (LoA = bias ± 1.96 SD) indicate the expected range for 95% of the individual differences across throws.

Agreement analysis between IMU and coach-based measurements showed small average biases across phases, with mean differences below 0.03 s. Mean absolute errors ranged between 0.01 and 0.04 s, and root mean square errors (RMSE) were consistently below 0.05 s. However, the limits of agreement revealed that coach annotations can deviate up to ±0.07 s (that is more than 8 IMU samples recording at 120 Hz), largely due to frame-selection uncertainty reflecting the inherent variability in coach-based video labeling. The times measured by the coach are not absolute values, for example, for the beginning/end of each phase, there were 3–4 frames (at a rate of 120 fps) that could be selected. Consequently, the mid-frame of these possible frames was selected to define the beginning or end of a phase, resulting in an average error of 0.0163 s in the times defined by the coach. In particular, the selection of the starting frame for the beginning of the double-support phase, *t_start_*, is especially complex (see Figure 1), where a group of 10–12 frames seemed to be static, making it difficult to ascertain in which one of them the phase starts.

In contrast, IMU sensors provide objective peaks in acceleration with a temporal resolution of 8.3 ms (120 Hz), yielding more consistent intra-athlete patterns (SD < 1% in athlete C in Table 2). These results confirm that IMU-derived measurements are closely aligned with coach annotations while providing a narrower range of uncertainty and higher temporal consistency.

### 3.4. Validation of the Procedure with a Female Athlete

**Figure 17:**
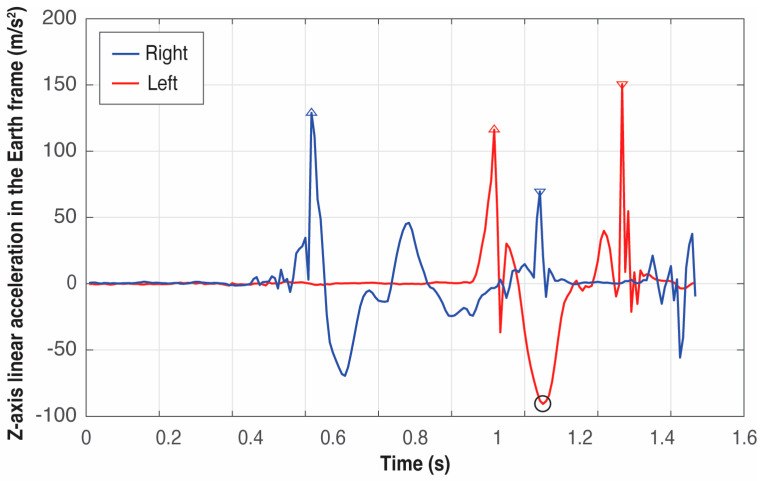
Detection of the four critical events of the left (red) and right (blue) feet in one trial of the female athlete. The triangles pointing upwards represent the takeoff of the feet and the triangles pointing downwards represent the landing. The circle in black represents the global minimum in the *Z*-axis of the left foot used to define the four critical events.

Due to physiological differences between male and female throwers and variations in implement dimensions, the procedure was evaluated with one female athlete across six trials. The objective of these experiments was to determine whether the acceleration patterns generated by the athlete’s feet were comparable to those of male throwers, thus allowing the algorithm to be applied without modifications. As demonstrated in Figure 17, where one of the trials is shown in detail, the observed acceleration patterns clearly parallel those recorded in male participants.

## 4. Discussion

The application of inertial sensors in rehabilitation exercises and performance analysis in sports with many practitioners (i.e., skiing, swimming, tennis, cycling, football, etc.) has been a continuous subject of research for the last two decades. Consequently, there are already many commercial products based on these electronic devices (maybe the most well-known are smartwatches for refining the execution techniques in swimming or golf). However, in minoritarian sports disciplines, such as the athletics throwing events, IMUs have not yet achieved the same level of popularity. However, they are gaining increasing attraction and interest because they have become more affordable, smaller and more sophisticated consumer-grade products, making them accessible to coaches, sports researchers, and athletes.

Moving the narrative to the field of the throwing events, although it is easy to extract and visualize orientation angles, linear accelerations, and angular velocities from commercial IMU-based solutions, it is necessary to interpret the signals to detect the relevant events from a biomechanical point-of-view according to the specific athletic movement. Due to the precise timing that IMUs can provide (in the order of milliseconds) and the complexity and variability of the signal patterns, it is clearly necessary to define the procedure for identifying these events of interest. Only through such a process can the obtained data provide a comprehensive framework for kinematic diagnosis of throwing motions. In our opinion, this paper presents a kick-off step for the consecution of this objective, at least, in the context of the discus discipline.

The validation of the findings using IMU with other similar procedures has not been possible. All the references in the literature where an analysis of the phases is presented are always based on data extracted by coaches or sport engineers from the analysis of video recordings.

Regarding the comparison of our results with data from video-based procedures, Figure 14 shows that there is consistency in the durations of the separate phases across the different studies. However, small differences may be attributable to the limitations in the current study. The number of Spanish participants was reduced, *M* = 3, and the participants’ performance levels differ from those observed in other studies. In our study, the three athletes compete in the Spanish National Absolute Championships rather than in World Athletics or Olympic Championships, in contrast to the other studies. In addition, the experiments were conducted during regular training but not in official competitions where athletes reach their performance peaks. However, if only data from athlete C are considered, his results are analogous to those from the other sources because this athlete presents a performance peak and skills similar to the elite participants in other sources. In any case, the measurements of the IMU-based procedure are consistent and aligned with the data obtained from these other sources.

Concerning the robustness of the algorithm, the detection of the start and end time instants of the throwing movement is not subject to false positives due to the method applied for the detection of both events. Regarding the four events associated with the feet, a common pattern in the maxima associated with the takeoffs and touchdowns was identified after having analyzed all the trials. Thus, if the procedure is applied as explained before (first, detection of *t_release_* and *t_start_*; second, detection of the global minimum in the *Z*-axis of the left foot; third, detection of the two takeoff times; and forth, detection of the two touchdown times), false positives should not occur when the procedure is applied to other male athletes with established discus throwing techniques.

Regarding the comparison between data from IMUs and from video recordings, although the mean differences between IMU and coach-based measurements were small, the limits of agreement demonstrated that coach annotations can deviate up to ±0.07 s, equivalent to more than eight IMU samples at 120 Hz. This discrepancy is largely attributable to frame-selection uncertainty, since coaches must choose among several visually plausible frames when identifying foot contacts or discus release. In contrast, IMUs detect these events automatically from acceleration peaks with an objective temporal resolution of 8.3 ms per sample. Moreover, IMU measurements showed very low intra-athlete variability (SD < 1% in athlete C, for example), underscoring their repeatability and robustness. Taken together, these findings indicate that IMU-based detection is not only valid when compared with coach-based labeling, but also superior in precision and reproducibility, providing a more reliable reference for biomechanical and coaching applications. Despite all the above, consumer-grade IMUs can also present certain issues, particularly for the acquisition of kinematics parameters of elite throwers. Most IMUs can measure up to 16 g and 2000°/s, but some athletes may exceed these limits (for example, Olympic javelin throwers can generate implement spins up to 25 Hz or 9000°/s [14]). In these situations, high performance IMUs and specific electronic designs are required.

## 5. Conclusions

The methodology proposed in this study demonstrates that commercially available IMUs provide a reliable, accurate, and unambiguous solution for detecting the critical temporal events in discus throwing. Our comparison with video-based measurements obtained by professional coaches validates that this procedure eliminates the frame-by-frame uncertainty inherent in traditional video analysis methods. The 100% detection rate without false positives across all trials confirms the robustness of our approach.

The procedure described in the paper is designed to be independent of the athlete profile because the feet’s acceleration patterns of the elite throwers (that is, the presence of maxima and minima in the linear accelerations) are always similar with small differences, even between males and females. Additionally, the IMU-based detection method overcomes several limitations of video-based approaches:

It eliminates the subjectivity in determining exact frame contacts, particularly problematic with feet touchdowns where cushioning effects create visual ambiguity.

It removes the need for complex pre-calibration work typically required for motion capture systems.

It enables automatic detection through well-defined algorithms rather than manual frame annotation.

This research represents an important step toward democratizing advanced biomechanical analysis in athletics. By leveraging affordable consumer technology, the significant gap between laboratory-based research and practical field applications can be bridged. The established procedure provides both a research tool for sports scientists and a practical training aid for coaches and athletes seeking to optimize throwing technique through precise temporal analysis.

Building on this foundation, future research should focus on realizing additional trials with more male and female athletes integrating additional IMU placements to capture more comprehensive biomechanical parameters, correlating phase detection with performance metrics to identify optimal temporal patterns and adapting the methodology to other rotational throwing events (hammer throw and rotational shot put).

## Figures and Tables

**Table 1:** Characteristics of the three Spanish elite male discus athletes.

Athlete	Age (Year)	Height (m)	Body Mass (kg)	PB (m)
A	23	1.98	110	61.15
B	24	1.94	105	56.77
C	26	1.98	107	65.54
Mean ± SD	24.33 ± 1.52	1.96 ± 0.02	107.33 ± 2.51	61.15 ± 4.38

**Table 2:** Mean and standard deviation (SD) of the duration of the phases for each of the athletes, *N* the number of throws studied for each athlete.

Athlete	A (*N* = 8)	B (*N* = 8)	C (*N* = 6)	All (*N* = 22)
	Mean ± SD	Mean ± SD	Mean ± SD	Mean ± SD
Total throwing time (s)	1.553 ± 0.031	1.603 ± 0.028	1.425 ± 0.021	1.536 ± 0.078
Duration of the phases				
Double-support phase (s)	0.548 ± 0.031	0.686 ± 0.017	0.586 ± 0.012	0.609 ± 0.065
Single-support phase (s)	0.392 ± 0.048	0.431 ± 0.034	0.339 ± 0.015	0.391 ± 0.051
Non-support phase (s)	0.139 ± 0.033	0.143 ± 0.033	0.121 ± 0.011	0.135 ± 0.029
Second single-support phase (s)	0.252 ± 0.026	0.162 ± 0.033	0.187 ± 0.010	0.202 ± 0.047
Delivery phase (s)	0.222 ± 0.024	0.180 ± 0.011	0.192 ± 0.007	0.198 ± 0.024
Percentage of the duration of the phases				
Double-support phase (%)	35.279 ± 1.825	42.824 ± 0.970	41.128 ± 0.434	39.617 ± 3.634
Single-support phase (%)	25.219 ± 3.001	26.894 ± 1.924	23.776 ± 0.851	25.434 ± 2.454
Non-support phase (%)	8.976 ± 2.059	8.906 ± 2.098	8.485 ± 0.865	8.817 ± 1.761
Second single-support phase (%)	16.248 ± 1.804	10.137 ± 2.062	13.157 ± 0.679	13.183 ± 3.119
Delivery phase (%)	14.277 ± 1.434	11.239 ± 0.593	13.453 ± 0.579	12.948 ± 1.656

**Table 3:** Summary of the studies carried out in the five references used for comparison purposes.

Reference	*N*	Mean Distance	Equipment	Sampling	Procedure to Detect the Critical Instants
[22]	6	52.4	Analog camera/s	−	Visual inspection of the clip
[22]	17	64.01	Analog camera/s	−	Visual inspection of the clip
[20]	51	58.5	2 S-VHS cameras	60 fps	21 body landmarks manually digitized Qualitatively by visual inspection of the video clip
[24]	7	65.09	1 digital camera	300 fps	No body landmarks Qualitatively by visual inspection of the video clip frame by frame
[6]	8	42.08	2 digital cameras	120 fps	19 body landmarks manually digitized Qualitatively by visual inspection of the video clip

**Table 4:** Comparison of the duration of the phases measured by IMUs and a professional coach for two throws by athletes A and C.

Duration of the Phases	Measured by IMUs	Measured by a Professional Coach Using Video	Difference
Athlete A			
Double-support phase (s)	0.5333	0.604	−0.0707
Single-support phase (s)	0.3917	−	−
Non-support phase (s)	0.1417	0.491 ^*^	0.0424 ^*^
Second single-support phase (s)	0.250	0.216	0.034
Delivery phase (s)	0.2163	0.225	−0.0087
Total throwing time (s)	1.533	1.537	−0.004
Athlete C			
Double-support phase (s)	0.5750	0.550	0.025
Single-support phase (s)	0.3167	0.325	−0.0083
Non-support phase (s)	0.1416	0.125	0.0166
Second single-support phase (s)	0.1917	0.2	−0.0083
Delivery phase (s)	0.192	0.183	0.009
Total throwing time (s)	1.417	1.384	0.033

* The beginning of the single support phase was not recorded by the coach. The difference between the cumulative time of single-support and non-support phases is shown.

**Table 5:** Agreement statistics between IMU and coach/video phase durations. Bias = mean difference (IMU-Coach), SD = standard deviation of the differences, MAE = mean absolute error, RMSE = root mean square error, CI95 = 95% confidence interval of the bias, LoA = limits of agreement (bias ± 1.96·SD).

Phase	DS	1SS	F	2SS	D	Total
Bias	−0.0229	0.0042	0.0211	0.0129	0.0001	0.0145
SD	0.0677	0.0177	0.0064	0.0299	0.0125	0.0262
MAE	0.0478	0.0125	0.0211	0.0212	0.0089	0.0185
RMSE	0.0530	0.0132	0.0216	0.0247	0.0089	0.0235
CI95_low	−0.6308	−0.1546	−0.0367	−0.2559	−0.1123	−0.2206
CI95_high	0.5851	0.1630	0.0790	0.2816	0.1126	0.2496
LoA- Low	−0.1555	−0.0304	0.0085	−0.0458	−0.0244	−0.0368
LoA_high	0.1098	0.0388	0.0338	0.0715	0.0247	0.0658

## Data Availability

This study involved biomechanical data gathered using inertial measurement units (IMUs) from athletes during discus throw training sessions. All participants are adults and provided written informed consent after being briefed on the study objectives, procedures, minimal risks (potential discomfort from sensor placement), and their right to withdraw at any time. Data were collected anonymously and stored securely in compliance with data protection regulations. The protocol was designed to minimize interference with athletes’ normal training routines and was reviewed by professional coaches to ensure no additional physical risks beyond those inherent to the sport.
